# Diagnosis and treatment of a 16-year-old Chinese patient with concurrent hereditary hemochromatosis and Gilbert's syndrome

**DOI:** 10.1186/s40001-014-0051-y

**Published:** 2014-09-28

**Authors:** Xianbo Wang, Yanmin Liu, Yujuan Chang, Huimin Liu, Peng Wang

**Affiliations:** Center of Integrative Medicine, Beijing Ditan Hospital, Capital Medical University, No. 8 Jing Shun East Street, Beijing, 100015 China; Department of Pathology, Beijing Ditan Hospital, Capital Medical University, No. 8 Jing Shun East Street, Beijing, 100015 China

**Keywords:** Hereditary hemochromatosis, Gilbert’s syndrome, Liver biopsy, Mutation

## Abstract

Gilbert’s syndrome and hereditary hemochromatosis predominantly affect Caucasians with a low incidence in Asians. Here we report the case of a 16-year-old Chinese boy, who was admitted with hepatalgia, jaundice, hyperpigmentation, and splenomegaly to our hospital. After excluding chronic hepatitis, autoimmune disorders, and alcohol or drug injury, genetic analyses of the patient and his parents revealed simultaneous manifestations of Gilbert’s syndrome and hereditary hemochromatosis, though his parents did not develop related symptoms. The presented case indicates that diagnoses of Gilbert’s syndrome and hereditary hemochromatosis should be taken into consideration when chronic hepatitis is suspected without a clear etiology.

## Background

Hereditary hemochromatosis (HHC) is an autosomal recessive genetic disorder caused by mutations in the high iron Fe (*HFE*) gene, which leads to exacerbated iron uptake and storage [1]. Gilbert’s syndrome (GS) is an autosomal inherited disease that manifests as intermittent unconjugated hyperbilirubinemia. It is caused by mutations of the UDP-glucuronosyltransferase1A1 (*UGT1A1*) gene, which has the function of rendering bilirubin water soluble via glucuronidation [2]. Because of the low HHC prevalence in Asians [3,4] and the high rate of GS unawareness [5], adolescent HHC concurrent with GS cases are rarely found in China.

## Case presentation

**Figure 1:**
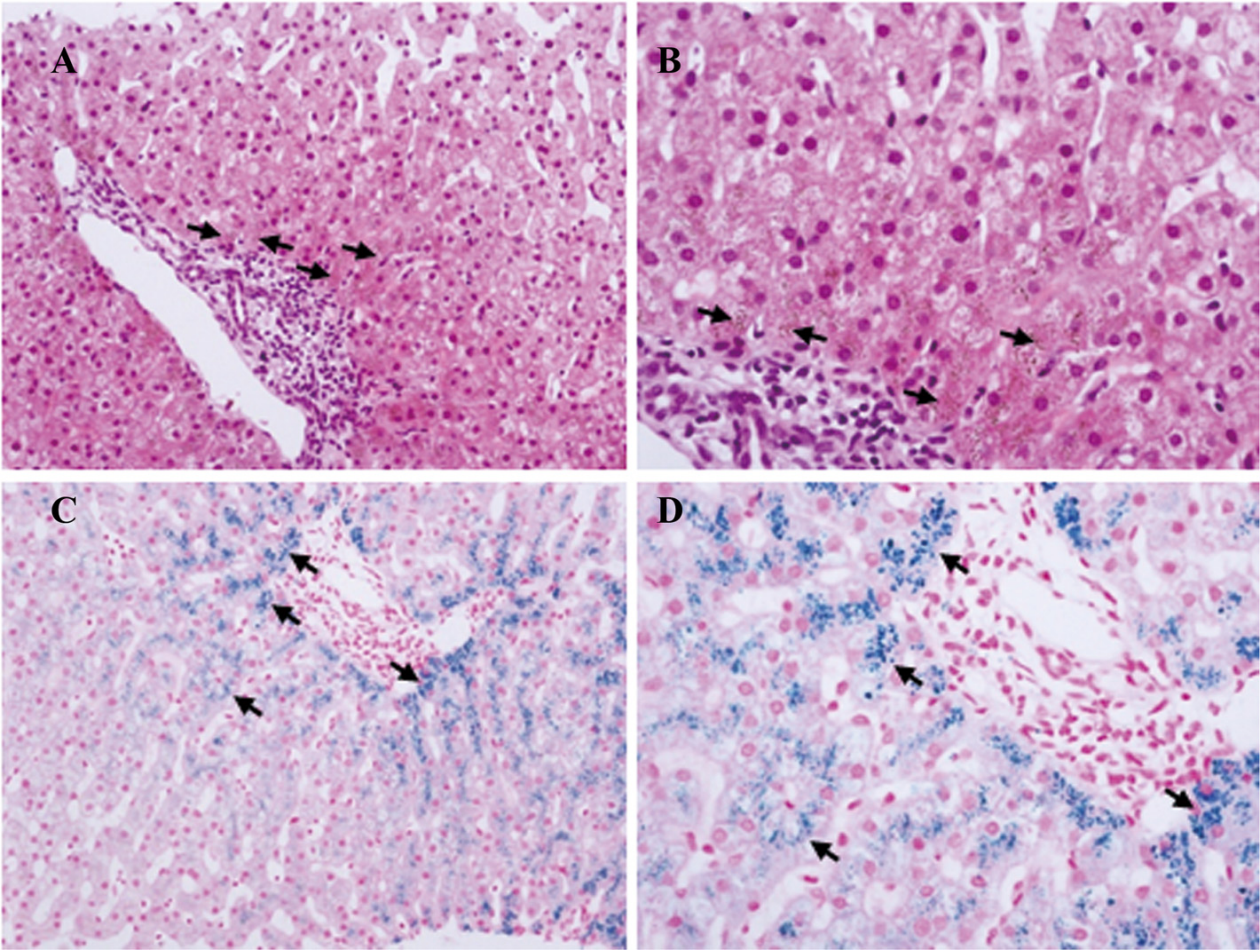
**Hepatic biopsy tissues (A and B: hematoxylin-eosin staining showing lipofuscin sedimentation (arrows) in acinar zone 1 hepatocytes.** **C** and **D**: prussian blue staining showing iron deposits (arrows) in the peripheral hepatic cells of the central vein. Original magnification: A and C ×200; B and D, ×400).

A 16-year-old boy was admitted to our hospital with a 1-year history of repeated epigastric pain and 6 months mild jaundice. We found skin hyperpigmentation, liver tenderness, splenomegaly, and mild jaundice of the skin and sclera during physical examination, while the first onset of skin hyperpigmentation occurred 3 years ago. We first presumed that he might have developed chronic hepatitis, caused by virus infections, drug or alcohol abuse, or autoimmune/metabolic disorders. Laboratory tests revealed 61.8 μmol/L total bilirubin (TBIL) (normal range, 0 to 18.8 μmol/L) and 7.5 μmol/L direct bilirubin (DBIL) (normal range, 0 to 6.8 μmol/L) concentrations, pointing to Gilbert’s syndrome. Iron metabolism analyses indicated abnormal serum concentrations of iron (39.42 μmol/L; normal range, 9.5 to 29.9 μmol/L), ferritin (410.02 ng/mL; normal range, 21.81 to 274.66 ng/mL) and transferrin (187.00 mg/dl; normal range, 200 to 400 mg/dl) (Table 1). Other serological parameters were normal (Table 1). An abdominal ultrasound examination revealed diffused liver lesions and splenomegaly (28 mm below the costal margin) but no abnormalities in the extrahepatic bile ducts or pancreas. Negative hepatitis serology, autoimmune antibody, and copper-protein results meant that viral hepatitis, autoimmune hepatitis, and Wilson’s disease could be excluded from the diagnosis. Because the patient did not consume alcohol or take drugs, did not receive blood transfusions or iron-supplementary nutrition, or ingest hepatotoxic drugs or chalybeate (natural mineral springs containing iron salts), we could also exclude secondary hemochromatosis, alcoholic hepatitis, and drug-induced hepatitis. In order to clarify the cause of the liver injury, a liver biopsy was carried out and the results revealed lipofuscin sedimentation in hepatocytes in zone 1 of the hepatic acinus (Figure 1A,B) as well as excessive iron in acinar zone 3 hepatocytes around the central vein (Figure 1C,D). The histopathological findings made us take a diagnosis of hereditary hemochromatosis (HHC) into consideration, though his family did not have a history of obvious HHC or disorders of iron metabolism. Gene analysis (SeqMan DNA Star 6.0 software, Lasergene, Madison, WI, USA) after amplifications with specific primers (Applied Biosystems, Carlsbad, CA, USA) revealed a heterozygous H63D and a homozygous IVS 2 + 4 T → C mutation in *HFE* as well as a heterozygous G71R mutation in the *UGT1A1* gene, confirming the diagnosis of HFE-related HHC and concurrent GS. After the definite diagnosis, the patient was discharged and a 300 mL therapeutic phlebotomy performed each week. However, after 9 weeks, the TBIL, iron, and ferritin serum levels of the patient were slightly enhanced (Table 2). We adjusted the bloodletting protocol to 400 mL per week and urged the patient to attend the clinic for phlebotomies more regularly. After 9 weeks, the relevant serum concentrations were decreased significantly and 4 weeks later, the TBIL and iron levels were reduced further (Table 2), with the patient’s symptoms being obviously relieved.

**Table 1:** **The baseline characteristics of the patient**

**Laboratory parameters**	**Results**	**Normal range**
Iron (μmol/L)	39.42	9.5-29.9
Transferrin (mg/dL)	187	200-400
Ferritin (ng/mL)	410.02	21.81-274.66
Alanine aminotransferase (ALT) (U/L)	8.4	9.00-50.00
Aspartate aminotransferase (AST) (U/L)	15.1	15.00-40.00
Total bilirubin (TBIL) (μmol/L)	61.8	0-18.80
Direct bilirubin (DBIL) (μmol/L)	7.5	0-6.80
Total protein (TP) (g/L)	76.9	65.00-85.00
Albumen (ALB) (g/L)	49.4	40.00-55.00
Globulin (Glo) (g/L)	27.5	20.00-40.00
Gamma-glutamyl transferase (GGT) (U/L)	12.8	10.00-60.00
Alkaline phosphatase (ALP) (U/L)	110.3	45.00-125.00
Cholinesterase (CHE) (U/L)	6882	4000.00-11000.00
Total bile acid (TBA) (μmol/L)	3.6	0-10.00
Prothrombin activity (PTA) (%)	68.3	80-120
White blood cell (WBC) (10^9^/L)	8.79	4.00-10.00
Neutrophilic (%)	62.5	50.00-75.00
Lymphocytes (%)	31.2	20.00-40.00
Red blood cell (RBC) (10^12^/L)	3.71	4.00-5.50
Hemoglobin (HB) (g/L)	133.4	120.00-160.00
Platelet (PLT) (10^9^/L)	290	100.00-300.00
Anti-nuclear antibody (ANA)	Negative	Negative
Anti-mitochondria antibody (AMA)	Negative	Negative
Anti-smooth muscle antibody (SMA)	Negative	Negative
Human anti-gastric parietal cell antibody (AGPA)	Negative	Negative
Human anti-myocardial antibody (HMA)	Negative	Negative
Human anti-liver-kidney microsomal antibody (LKM)	Negative	Negative
Human anti-mitochondrial antibody M2 subtype (AMA-M2)	Negative	Negative
Human anti-centromere antibody (ACA)	Negative	Negative
Immunoglobulin G (g/L)	12.7	7.51-15.60
Immunoglobulin A (g/L)	2.02	0.82-4.53
Immunoglobulin M (g/L)	1.49	0.40-2.74
Complement 3 (C3) (g/L)	0.53	0.79-1.52
Complement 4 (C4 ) (g/L)	0.13	0.16-0.38
Ceruloplasmin (g/L)	0.21	0.22-0.58
Rheumatoid factor (RF) (IU/mL)	<20	0-20.00
α1-Globulin (g/L)	3.6	2.9-4.9
α2-Globulin (g/L)	5.3	7.1-11.8
β1-Globulin (g/L)	4.3	4.7-7.2
β2-Globulin (g/L)	2.9	3.2-6.5
γ-Globulin (g/L)	16.6	11.1-18.8
Hepatitis B surface antigens (HBsAg) (IU/mL)	Negative	<0.05
Anti-HCV (S/CO)	0.11	<1
Blood glucose (μmol/L)	5.09	4.16-6.44

**Table 2:** **Changes of ferrokinetic parameters and bilirubin levels in the patient’s serum during bloodletting therapy**

**Follow-up**	**Bloodletting therapy**	**TBIL (μmol/L)**	**DBIL (μmol/L)**	**Serum iron (μmol/L)**	**Transferrin (mg/dL)**	**Ferritin (ng/mL)**
0	0	61.80	7.50	39.42	187.00	410.02
1	300 mL/week	105.60	9.80	40.07	155.00	424.19
2	400 mL/week	41.70	7.40	7.97	224.00	324.51
3	400 mL/week	37.20	7.60	8.42	234.00	333.68

The research was approved by the ethics committee of Beijing Ditan hospital, Capital Medical University.

## Discussion

The low prevalence in China and non-specific symptoms can mean that the diagnosis of HHC might sometimes be overlooked. However, as the patient developed hyperferritinemia, we suspected hemochromatosis and since he had not previously had blood transfusions or ingested chalybeate, secondary hemochromatosis could be excluded. However, for a definite HHC diagnosis, genetic testing is necessary and we also genetically screened the patient’s first-degree relatives. His mother was found to be a homozygous IVS 2 + 4 T → C and heterozygous *UGT1A1* G71R mutation carrier without symptoms. His father was homozygous for IVS 2 + 4 T → C and has a heterozygous H63D mutation. His *UGT1A1* gene contains a heterozygous G71R and an additional heterozygous Y486D mutation, which he did not pass over to his son.

Some authors suggest that IVS 2 + 4 T → C might be involved in specific splice activity [6] but no correlation with HHC or the incidence of iron overload could be detected [7,8], which explains the lack of HHC symptoms in the patient’s mother. In contrast to Caucasians, HHC is rare in Asians and mostly restricted to H63D mutations in the *HFE* gene [9,10]. A H63D heterozygous mutation is not generally associated with a hemochromatosis phenotype unless it occurs as a C282Y-H63D compound heterozygosity, or additional exogenous factors are triggered. Although symptoms of obvious iron overload were absent in the father, his serum iron concentration of 33.44 μmol/L was slightly elevated and the 198 mg/dL transferrin value was close to the lower limit of the normal range.

G71R is the most common single nucleotide polymorphism (SNP) of the *UGT1A1* gene in Asia. Hepatic glucuronidation activity in subjects with heterozygous G71R mutations were 60.2 ± 3.5% of the wild type activity, which was considered somewhat high for a subject to develop GS [9] and is in accordance with the normal bilirubin serum concentration of the patient’s mother. In contrast, Y486D SNP were reported to have stronger inhibitory effects on *UGT1A1* and most Asian GS patients belong to the compound G71R/Y486D mutation group [10,11]. The data of the patient’s father are in line with these findings because his *UGT1A1* gene contains heterozygous G71R plus Y486D mutations with resulting enhanced bilirubin levels. Extraordinarily, our 16-year-old patient, who has only one heterozygous *UGT1A1* SNP (G71R), developed even higher TBIL and DBIL serum concentrations (Table 3).

**Table 3:** **Bilirubin levels and ferrokinetic parameters in the family**

	**TBIL (μmol/L)**	**DBIL (μmol/L)**	**IBIL (μmol/L)**	**Serum iron (μmol/L)**	**Transferrin (mg/dL)**	**Ferritin (ng/mL)**
The patient	61.80	7.50	54.30	39.42	187.00	410.02
His father	27.20	9.40	17.80	33.44	198.00	44.78
His mother	14.80	3.00	11.80	13.27	246.42	91.80

Normal ranges: TBIL, 0-18.8 μmol/L; DBIL, 0-6.8 μmol/L; serum iron, 9.5-29.9 μmol/L; transferrin, 200-400 mg/dL; ferritin, 21.81-274.66 ng/mL.

*HFE* mutations alone do not automatically produce symptoms of iron overload. Iron stores and inflammation are inducers of hepcidin, which acts as negative regulator of iron absorption in the small intestine as well as iron release from macrophages [12]. Impairment of hepcidin activity, which is principal regulator of systemic iron homeostasis, has been correlated with HFE-related hemochromatosis [13]. Also, bilirubin levels are thought to influence the state of iron homeostasis. An earlier publication noted that 23% of HHC patients showed increased unconjugated bilirubin serum concentrations [14]. Bilirubin is an antioxidant [15] and iron causes oxidative stress [16]; oxidative stress-induced hemeoxygenase-1 activity has been proposed to cause enhanced bilirubin serum concentrations in some *HFE* mutation carriers, which counteracts the high oxidative stress produced by excess iron and may thus contribute to reduced penetrance [17]. The protective role of bilirubin in individuals with *HFE* mutations might be applicable to the patient’s father, but based on observations of patients with HHC and simultaneous mutations of the *UGT1A1* gene, Romanowski *et al.* proposed that high levels of bilirubin might promote iron loading by decreasing oxidative stress, in addition to reduced hepcidin signaling due to less inflammatory processes [18], which might explain the iron overload in the son. Notably, the son developed more pronounced enhancements of TBIL and IBIL serum concentrations compared to his father, even though he has one less mutation in his *UGT1A1* gene. We suggest that in this case, increased iron absorption lead to oxidative stress and induced heme oxygenase, resulting in an increase in serum bilirubin. The higher bilirubin levels led to higher iron storage tolerance and in turn triggered even more heme oxygenase activity. Finally, the balance between oxidative stress and antioxidant bilirubin production caused the pronounced elevated bilirubin and serum iron concentrations.

## Conclusions

In summary, our 16-year-old Chinese patient with hepatalgia, jaundice, hyperpigmentation, and splenomegaly was shown to be a heterozygous H63D and homozygous IVS 2 + 4 T → C *HFE* and heterozygous G71R *UGT1A1* gene mutation patient with simultaneous HHC and GS manifestations. Astonishingly his father, who had the same SNPs in both genes plus an additional heterozygous Y486D mutation in *UGT1A1*, was not affected.

## Consent

Written informed consent was obtained from the patient’s parents for the publication of this report and any accompanying images.

## Abbreviations

DBIL: Direct bilirubin
GS: Gilbert’s syndrome
HHC: Hereditary hemochromatosis
IBIL: Indirect bilirubin
SNPs: Single nucleotide polymorphisms
TBIL: Total bilirubin
*UGT1A1*: UDP-glucuronosyltransferase 1A1
